# Study on Nutrient Carrier of Mulch Based on Hydrogel @SiO_2_

**DOI:** 10.3390/polym16101442

**Published:** 2024-05-20

**Authors:** Dan Qin, Yujie Ma, Mei Wang, Zhihua Shan

**Affiliations:** 1 School of Chemistry and Materials Science, Sichuan Normal University, Chengdu 610068, China; chin0142@163.com (D.Q.); chemical_ruby@sohu.com (Y.M.); 2 College of Biomass Science and Engineering, Sichuan University, Chengdu 610065, China; pengzhuo3@gmail.com

**Keywords:** protein polypeptide, poly vinyl alcohol, encapsulated soil granules, soil moisture conservation, soil fertility, plant water adsorption

## Abstract

Soil conservation is one of the best methods to improve soil fertility and enhance crop growth efficiency. Replacing plastic mulch with biomass is an environmentally friendly strategy. Innovative encapsulated soil granules (ESGs) were developed using PVA/PC film as the wall material and rural soil as the core. The PVA/PC was synthesized using 60% protein polypeptide (PC) from leather waste scrap and 35% poly (vinyl alcohol) (PVA), which was optimized for water absorption expansion and water retention performance. The ESG-10 granulated with 10% PVA/PC exhibited good water absorption, moisture retention, and resistance to water solubility. As an auxiliary material for soil improvement, the amount of ESGs mixed with the topsoil at ratios of 0 g/m^2^, 200 g/m^2^, and 400 g/m^2^ was proportional to the soil insulation and moisture retention. In rapeseed cultivation, the experimental results indicated that the soil mulched with ESG-10 can maintain seedling vitality for a long time under low water content conditions.

## 1. Introduction

Under the same climatic conditions and soil chemical composition, the physical structure of the soil, also known as aggregate structure, is the key factor determining plant growth [1,2,3]. Most dry areas are characterized by higher evaporation than precipitation, resulting in rapid water loss from the soil and its return to the atmosphere, leading to ineffective water cycling [4,5,6]. Therefore, developing technologies for soil moisture conservation, also known as soil water and temperature retention, is a top priority in soil-dependent farming. It is widely accepted that higher soil temperatures can accelerate seed germination and crop growth, leading to increased production. At the same time, it is beneficial for the accumulation of dry matter and more efficient water utilization in the early stages of crop growth. The practice of soil mulching involves placing a continuous coating over the soil, which is widely recognized for its role in improving soil moisture conservation [7,8,9,10]. Plastic films were first recognized for their ability to improve soil water and soil temperature, and it has been reported that they directly affect the soil microclimate by modifying surface radiation [11,12]. The yield of food and cash crops increased by 35% and 60%, respectively, after plastic mulching [13]. However, residual farmland films not only impact the ecological environment but also degrade the physical and chemical properties of the soil, resulting in low yields and environmental pollution [14,15]. As a result, relevant reports have been continuously published to address the problem of residual plastic film pollution and improve crop growth [16]. An innovative approach has been introduced in the form of biodegradable mulch films, which use natural or synthetic biodegradable polymers such as starch, proteins, cellulose, chitin, and chitosan as raw materials [17,18]. The use of biodegradable raw materials in mulching films is an excellent alternative to plastic films [19,20,21]. When diluted and sprayed on the surface, it forms a gel-like film that quickly condenses the dispersed soil particles into aggregated structures, thereby inhibiting water evaporation and warming the soil [22,23,24,25]. At the end of the crop growth cycle, the biodegradable materials can be directly integrated into the soil, where microorganisms transform them into carbon dioxide or methane, water, and small organic components [26,27,28,29]. This approach has broad application prospects for environmental protection and sustainable development. However, the mechanical properties of sprayable mulch need to be taken into consideration to retain integrity during its use and resist damage from environmental factors such as rainfall, high winds, high or low temperatures, and if possible, extraneous damage from wildlife [30,31,32]. Additionally, sprayable mulch is not a self-supporting material. The mechanical and other properties of sprayable mulch are influenced by its adhesion to and support from the soil, the cross-linking that may occur during drying, and the spatial variations in coating thickness [33,34,35]. The growth of crops is greatly influenced by the soil type, including factors such as soil surface uniformity, flatness, aggregate size, and climatic conditions. Therefore, the selection, application methods, and costs of mulching films still require further exploration and research [20,35,36,37].

Polypeptides based on collagen (PC) are well-known products composed of a mixture of collagen degradation products obtained from enzymatic or chemical hydrolysis of chrome tanned leather shavings from the tanning industry. As such, they are easily available at low cost and are highly biodegradable [20]. On the other hand, protein-based materials used as farm manure have a high nitrogen and carbon content, which increases their agronomic value when mixed with various materials to create biodegradable biomass nutritional mulch [34,35]. Poly (vinyl alcohol) (PVA) is a polymer obtained by hydrolysis of poly (vinyl acetate). It is soluble in water and is a hydrophilic synthetic polymer that can be blended with natural products due to its compatibility and film-forming ability. PVA offers excellent mechanical properties, barrier resistance to gases and aroma, water resistance, and non-toxicity. Furthermore, PVA has shown a positive effect on soil structure, making it suitable for application in open fields [25]. There have been numerous studies on the incorporation of natural polymers into PVA, such as hydrolyzed protein/PVA [7], soy protein isolate/PVA [14], myofibrillar protein/PVA, and others produced by the solution-casting method. Protein/PVA films have been investigated for applications in food packaging, protective films, edible films, and agricultural mulching films [38,39,40]. It has been demonstrated that a mixture of protein polypeptide and PVA forms mulch with excellent water retention and biodegradable properties [7].

In this study, we conducted an investigation to evaluate the possibility of developing an innovative biodegradable mulch in the form of encapsulation granules as an alternative to membrane covering, with the aim of improving soil water and temperature in coarse or uneven soil surfaces. In the mountains and areas with hard-to-cultivate soil, the use of encapsulation granules that roll into the soil cracks and then absorb water to expand and complete the coverage simplified the operation and saved the amount of mulch required. This approach has practical significance for planting in such soil environments. The encapsulation granules were composed of PVA/PC as the shell material and rural soil as the core material, referred to as PVA/PC@soil. When sprinkled on the surface of the soil by mechanical or manual means, the encapsulation granules automatically rolled into the gaps of the coarse soil and then expanded and occluded through water absorption. The experimental approach was first developed and tested in the laboratory, and then the pot culture was evaluated [20]. It was found that the new encapsulation granules had the effect of preserving soil moisture and temperature, and promoting the growth of vegetable seedlings. At the same time, this approach has special significance for reducing the use of plastic film mulching [41,42,43,44].

## 2. Materials and Methods

### 2.1. Main Materials 

Polypeptide powder based on collagen was provided by Xuzhou Hongfeng Polymer Materials Company, Xuzhou, Jiangsu, China. This powder was obtained through alkaline hydrolysis and extraction from leather waste scraps [11]. The main component of the PC was chromium 23.1 mg/kg, with ash at 49.7 mg/kg, and the molecular weight was 3.4~30 kD. 

Sodium hydroxide, polyvinyl alcohol (PVA), formaldehyde, and urea were all chemically pure and came from Chengdu Shudu Ltd., Chengdu, China. 

Rural soil (or pastoral soil) was from a farmland in Shandong, China (bought online). The pH of the 20% turbid liquid soil was 8.6. The water content was 0.6%. Soil ash content was 95.4% (it was placed in a muffle furnace at 750~850 °C for 5 h). The element composition of rural soil was examined by analyzer (Hitachi S-4800, Tokyo, Japan) and is shown in Table 1.

Rapeseed seed (germination state 80%) was purchased from the Nanwu Science Trade Co., Ltd., Beijing, China.

### 2.2. Preparation and Characterization of Hydrogel PVA/PC

An HS20-S laboratory digital display 150 W electric mixer (Zhengzhou Penglai Instrument Equipment Co., Ltd., Zhengzhou, China) was used to synthesize PVA/PC. We diluted PC powder to 16% concentration with deionized water, added it to a three-necked round-bottomed flask, placed it in a water bath and stirred at 50.0 ± 0.2 °C. The temperature of the reaction mixture was increased gradually up to 80 °C, at which point urea and PVA were incorporated and allowed to mix thoroughly under constant stirring. The adjusted pH was 8.0 with 2% sodium hydroxide solution and 5% formaldehyde as the crosslinker added continuously within 30 min. The reaction temperature was then increased to 85 °C and was maintained for one hour. Finally, the reaction product, PVA/PC solution, was placed in a 50 mm culture dish and dried in the air at 25 °C, 60~65% relative humidity (RH) after air drying to obtain a translucent PC/PVA film with a thickness of 1 mm. Table 2 shows the four materials with varying compositions prepared, which were encoded as PVA/PC-80, PVA/PC-60, PVA/PC-40, and PVA/PC-20, respectively. 

#### 2.2.1. Equilibrium Moisture Content of PVA/PC Films

Four kinds of films, called PVA/PC-X film, were placed in an instrument with constant temperature and humidity (DHG-9140A, Shanghai, China) at 25 ± 1 °C, RH = 60~65% for 24 h, and then the PVA/PC-X films were weighed (*W*_e_) and dried to a constant weight (*W*_0_) at 105 ± 1 °C. The equilibrium moisture content (*E*_moisture_) of each PVA/PC-X film was obtained. The experiment was repeated three times, and the average value was used.

(1) $$E_{\mathrm{moisture}}=\frac{W_{e}-W_{0}}{W_{0}}\times 100\%$$

#### 2.2.2. Water Solubility of PVA/PC-X Films

All sample films were immersed in a Petri dish filled with deionized water at 25 ± 1 °C for 12 h. The weight (W12) of films was tested when the surface moisture on the sample was absorbed by filter paper. The solubility of films was tested by W12 and W0 of each piece of sample (see Equation (2)). W12 and W0 were obtained at the constant weight of 105 °C, where W0 was the constant weight of another unsoaked sample.

(2) $$\mathrm{Solubility}=\frac{W_{0}-W_{12}}{W_{0}}\times 100\%$$

The experiment was repeated three times and the average value in (2) and (3) was used.

(3) $$A_{\mathrm{Water}}=\frac{W_{t}-W_{0}}{W_{0}}\times 100\%$$

#### 2.2.3. Water Absorption of PVA/PC-X Films

Each PVA/PC-X film that balanced the moisture content was cut into several pieces of about 2 g each, and its original weight W0 was obtained according to Equation (1). All sample films were immersed in a Petri dish filled with deionized water at 25 ± 1 °C for 12 h, during which the weight (*W_t_*) of films was tested at appropriate intervals when the surface moisture on the sample had been absorbed by filter paper. If not counting the dissolved parts the water absorption of film was calculated in Equation (2) and each PVA/PC-X film was weighed again until reaching constant weight (*Wt*−0) at 105 °C. The solubility according to W0 and Wt-0 of each piece of sample was tested, see Equation (3). The experiment was repeated three times and the average value in (2) and (3) was used.

#### 2.2.4. Water Loss of the PVA/PC-X Films

All PVA/PC-X films were immersed in a Petri dish filled with deionized water at 25 ± 1 °C for 12 h and weighed to obtain Wc values, respectively. We put the PVA/PC-X films in an instrument with constant temperature and humidity (DHG-9140A, Shanghai, China) at 25 ± 1 °C, RH = 60~65% and took them out every 3 h to weigh them and obtain the *Wc*−*t* values. The water loss in the PVA/PC-X films was calculated by Equation (4). The experiment was repeated three times and the average value was used.

(4) $$\mathrm{Water}\;\mathrm{loss}=\frac{W_{c}-W_{c-t}}{W_{c}}\times 100\%$$

#### 2.2.5. DSC Analysis of the PVA/CP-X Film

Pure PC and PVA were dissolved and prepared as films and together with PVA/CP-X films. The thermal properties of the three films were measured respectively by the differential scanning calorimetry analyzer (200PC, Germany NACH instrument manufacturing Co., Ltd., Duisburg, Germany). The operating parameters were a temperature rise rate of 5 ± 2 °C/min, protection nitrogen rate of 60 mL/min, and test temperature range of 30~100 °C. 

### 2.3. PVC/CP-X Encapsulated Soil Granules

**Figure 1:**
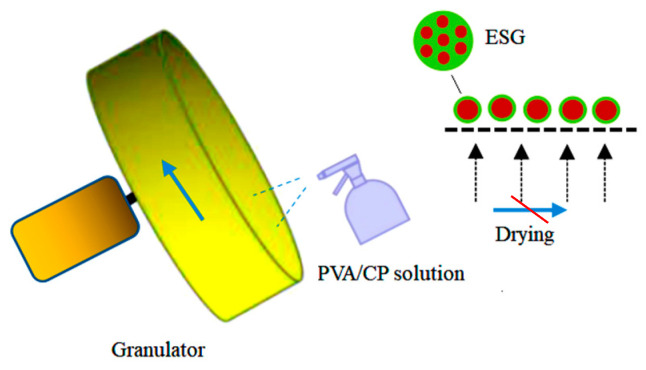
Scheme of applied granulation process.

The mixture of rural soil was transferred into the granulator (500 mm in diameter, 150 mm in width, and 35 r/min in speed). The soil granules’ diameter and density could be controlled by the rotation speed, time in the granulator, and soil moisture content of the mixture. Four different concentrations (5.0%, 10%, 15%, and 20%) of the optimal PVA/PC-X solution were gradually sprayed during the stirring of the granulator. About 20% solution weight (based on the soil particle weight) was sprayed onto the soil surface and the granule diameters were stabilized at 2.5~3.5 mm. Four concentrations of PVC/CP-X encapsulated soil granules (ESGs), a group of PVC/CP@Rural soil granules, were obtained and are simply labeled here as ESG-5, ESG-10, ESG-15, ESG-20, respectively. The spherical sample preparation process is shown in Figure 1.

#### 2.3.1. Water Absorption and Volume Expansion of ESGs

Four kinds of ESGs were immersed in water, and their volume expansion was observed. The water absorption of four ESG samples was tested according to experiments Section 2.2.2 and the expansion volumes were calculated according to ESG diameter. The optimal expanded ESG sample was selected to future research. 

In reference experiment Section 2.2.3, expanded ESGs were placed in a constant temperature and constant humidity chamber at 60~65% RH, 25 ± 1 °C. The evaporation process of the water in ESGs was calculated by weighing every 3 h. The selection of the best moisture retention samples was further explored experimentally. After a cycle was completed, it was left standing for 24 h at 60~65% RH, 25 ± 1 °C, then taken out again and immersed in water, and the maximum water absorption was measured. The relationship between the number of cycles and water absorption was obtained by repeating 4 times.

#### 2.3.2. Water Loss of the ESGs

Each loop method was 10 g of ESGs balanced at 25 ± 1 °C, RH = 60~65% for 24 h and put into a beaker containing 50 mL of distilled water at 22~24 °C in air for 24 h. The water absorption ratio and the expansion volume of ESGs were calculated by Equation (2).

#### 2.3.3. Temperature and Humidity Conservation of Soil Mulched by ESGs

The soil moisture conservation testing of the ESG mulching was performed in the open air. The temperature and moisture retention effect of ESGs was tested inside three glass pots (length 300 mm, width 400 mm, height 400 mm), which were filled with soil to a depth of 300 mm (about 41 kg dry soil each pot). The experimental comparison dosages of ESGs were set as 0 g/m^2^, 200 g/m^2^, and 400 g/m^2^ and labeled as ESG-0 (0 g/m^2^), ESG-200 (200 g/m^2^), and ESG-400 (400 g/m^2^) respectively. A sensor was inserted into each glass planting basin at a depth of 50 mm (depth of rapeseed planting). Before the test, the same amount of water was sprinkled three times at intervals of 4 h and the total amount of water was 10% of the soil weight in the pot (about 4 kg water). After this time, without water, fertilizer, and pesticides were applied during the process of rapeseeds germination and growth. The temperature and humidity of soil were recorded every 2 h. The optimized ESG mulching was determined.

## 3. Results and Discussion

### 3.1. Equilibrium Moisture Content and Solubility of PVA/PC-X Films

**Figure 2:**
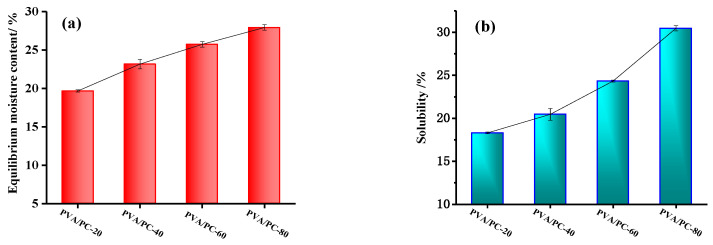
Equilibrium moisture content (**a**) and solubility (**b**) of four kinds of PVA/PC films.

The equilibrium moisture content of the PVA/PC-X films serves as a basis for their water retention ability. The equilibrium moisture content of polymeric mulch films in agriculture plays a crucial role in controlling their deterioration and degradation on and within the soil. The equilibrium moisture content of the four types of hydrogel PVA/PC-X films is presented in Figure 2. The experimental results showed significant differences in these PVA/PC-X films under the studied conditions. From PVA/PC-20 to PVA/PC-80, with an increasing of collagen content in the PVA/PC-X films, the hydrophilicity of the films and the equilibrium moisture increased. It was found that the equilibrium moisture of the PVA/PC-X films meant they had a higher water content than pure gelatin (≤14%, QB2354-98), so the PVA/PC-X films maintained a high water content at 25 ± 1 °C and RH = 60~65% after the addition of the PVA component. 

It has been proven that collagen and PVA possess strong water-binding abilities, but their self-assembly or self-aggregation can result in a loss of the efficacy of their hydrophilic groups. When collagen and PVA are evenly mixed and chemically crosslinked to achieve well-dispersed particles, the hydrophilic properties of the PVA/PC composite are optimized. Figure 2 demonstrates that increasing the amount of PC can enhance the moisture retention of the film, particularly when the PC content is around 40% (PVA/PC-40). This finding highlights the significance of achieving a balanced moisture level for improving soil moisture retention under the same humidity conditions.

However, Figure 2 also shows that the solubility values of the four PVA/PC-X films were 30.5% (PVA/PC-80), 24.3% (PVA/PC-60), 20.8% (PVA/PC-40), and 18.3% (PVA/PC-20), which indicates a rapid increase in solubility when the PC component exceeded 60%. In terms of practical application value, PVA/PC-60 hydrogel film is an ideal granulation wall material for the preparation of ESGs.

### 3.2. Water Absorption and Loss of PVA/PC-60 Film

**Figure 3:**
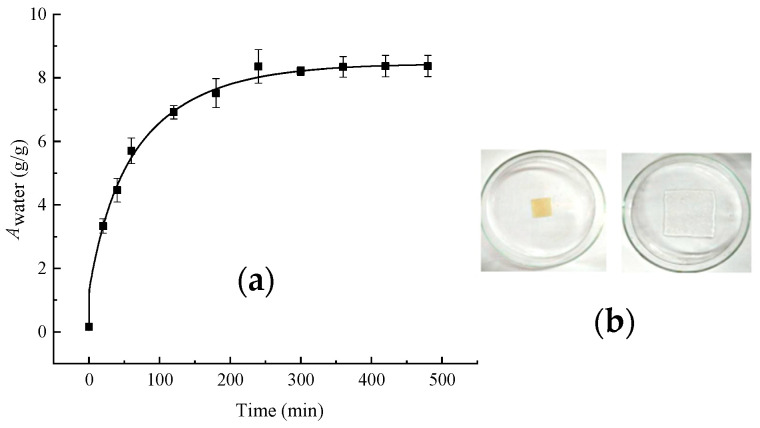
Awater-T curve (**a**) and expansion of PVA/PC-60 film (**b**).

Water absorption of the PVA/PC-60 hydrogel film was a prerequisite for achieving good ESGs in this study. The PVA/PC-60 film absorbs water and expands, which can increase its coverage. The stored water in the film is necessary not only for preserving soil moisture but also for reducing temperature fluctuations based on the high specific heat capacity of water. Furthermore, the growth of plants and microorganisms is inseparable. The results of the water absorption expansion of the PVA/PC-60 films are presented in Figure 3. After the PVA/PC-60 films absorbed water, the volume significantly increased, and the transparency was enhanced. The volume expansion ratio of PVA/PC-60 was 8.5.

**Figure 4:**
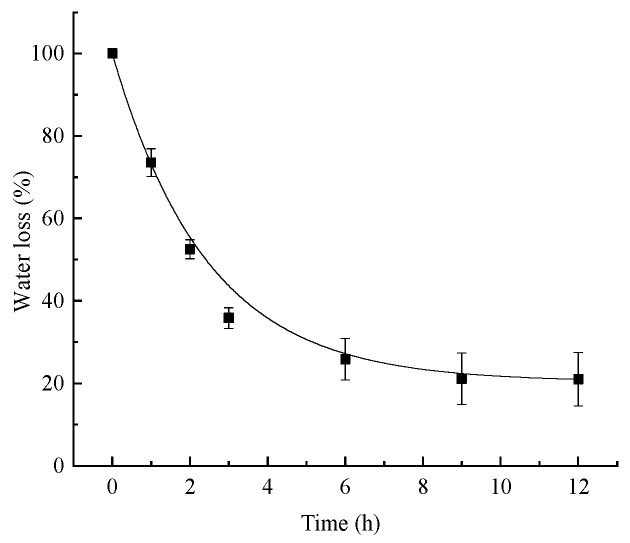
Water loss-T of PVA/PC-60 film.

Figure 4 shows the water loss of the PVA/PC-60 film at 25 ± 1 °C and a relative humidity of 60~65%. After approximately 3 h, the rate of water loss slowed down, indicating that the PVA/PC-60 film had a good water retention function.

### 3.3. The Softening Temperature of the Film

**Figure 5:**
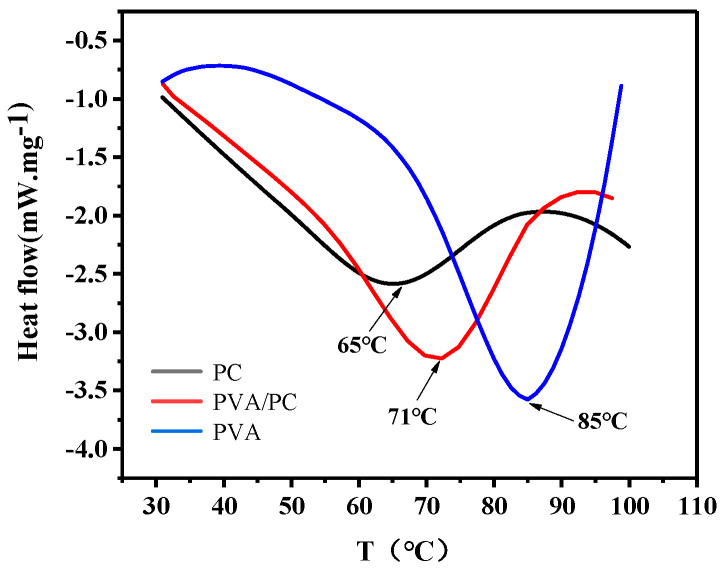
DSC analysis of films.

The softening temperature of the film is the temperature at which the film begins to deform and lose water, which affects the function of the mulching film as a covering in the atmosphere. DSC (Figure 5) is a commonly used method to measure the softening temperature of polymers. The softening temperature of the PVA/PC-60 film can be determined at the operating temperature, which affects its function as a mulching film in the atmosphere. On the one hand, the bound water in the film increases its heat absorption from 30 °C. On the other hand, compared to pure PC, the softening temperature of the PVA/PC-60 film is increased from 65 °C to 71 °C. The bound water mainly contributed by PC can significantly improve the stability of the PVA/PC-60 film as a mulching material in farmland during seasonal and climate changes.

### 3.4. Optimized Selection of ESGs 

**Figure 6:**
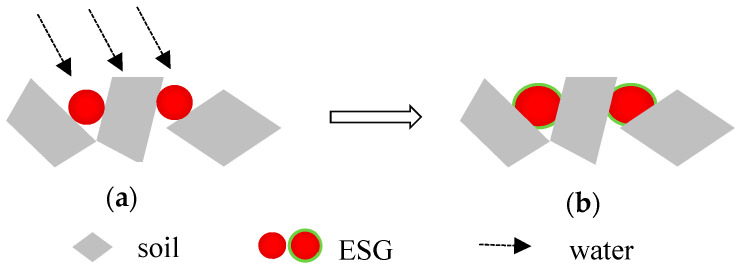
Performance of ESGs as a mulching film (Before rain (**a**), after rain (**b**)).

The water absorption and swelling of ESGs play a crucial role in soil thermal insulation and moisture retention, which can block the water evaporation of the soil pores. Additionally, under rainy conditions, it can reduce the impact of rain on the soil. The performance of ESGs as a mulching film is shown in Figure 6.

PVA/PC-60 was selected as the optimal wall material, and four ESG samples called ESG-5, ESG-10, ESG-15, and ESG-20 were prepared. Table 3 shows that the four samples absorbed water and expanded with time, and it can be seen from the table that both the water absorption rate and volume expansion rate of the ESGs decreased with increasing PVA/PC-60 concentration. Although increasing the amount of the PC in the PVA/PC-X film led to an increase in water absorption, gelatin served as a freeze–thawing agent in the ESGs composed of three components. As the amount of PVA/PC-60 increased, the internal crosslinking of PVA/PC/red soil increased, creating a tight network structure that hindered the entry of water and resulted in a decrease in the water expansion of the ESGs.

**Figure 7:**
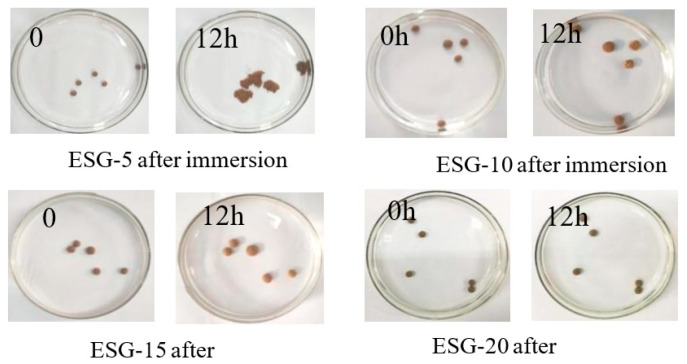
Shape change of ESGs during immersion period.

Figure 7 shows that ESG-5 was completely destroyed and lost its mulching function after being immersed for 12 h. ESG-10, ESG-15, and ESG-20 all demonstrated good resistance to water dissolution, indicating that the water resistance of the PVA/PC envelope can maintain the volume stability of ESGs. As can be seen from Figure 6, with the increase of the PVA/PC-60 solution concentration in the granulation process, such as in ESG-20, the water resistance of the ESGs was enhanced. The results indicate that ESG-10 and ESG-15 are promising candidates for further study, whether considering water absorption or the expansion multiple.

### 3.5. Water Retention of ESGs

**Figure 8:**
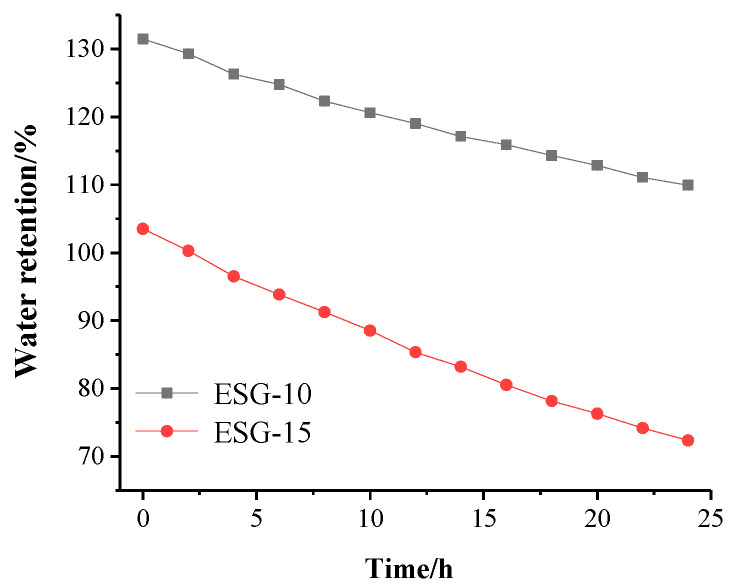
Water retention over time using ESGs.

Figure 8 presents the results of water retention (or water loss) of expanded ESGs at 25 ± 1 °C and RH = 60~65%. For ESG-10 and ESG-15, the moisture content of the samples significantly decreased. However, as the concentration of the PVA/PC-60 solution increased in the granulation process, the reduction in moisture content slowed down. This can be explained by the fact that ESGs effectively reduce the amount of soil water that can be evaporated. The better the water retention ability of ESGs, the more luxuriant the crop growth becomes. In other words, ESGs can be used as a water absorbent and retention material, which will be beneficial for improving the crop growth effectively. It is also conceivable that the PVA/PC-60 solution, with a concentration of 10~15%, is feasible for application in agricultural production. Compared to other options, ESG-10 was found more suitable as a mulching film and was the optimized choice.

**Figure 9:**
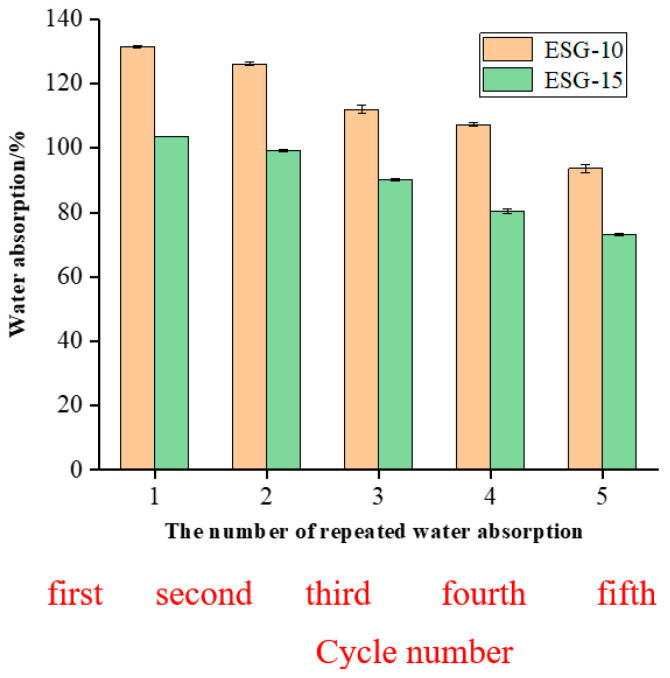
Cycles and maximum water absorption of two ESGs.

Plant growth occurs in alternating dry and wet soil environments. To meet this requirement, ESGs need self-repair capabilities, which means they must go through water absorption, expansion, evaporation, and water absorption again. However, as shown in Figure 9, the self-repair effect of ESG-10 and ESG-15 decreased with the increase of cycles.

This can be explained by the fact that during the repeated drying and soaking process, the solubility of the PVA/PC-60 increased or the degree of mineralization increased, resulting in a decrease in the water absorption rate. After five repeated tests, the water absorption ratios of ESG-10 and ESG-15 decreased from 93.7% and 73.2% to 28.7% and 29.3%, respectively. This showed that ESGs could be well recycled as a mulching film. In further experiments, ESG-10 was used as the optimized sample, designated ESG_0_, for mulching granule applications.

### 3.6. Moisture Conservation of ESGs

**Figure 10:**
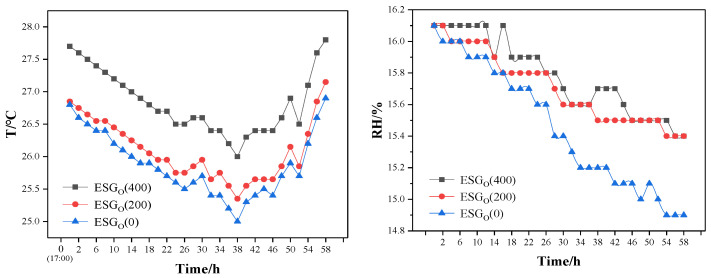
The temperature and humidity of soil mulched by ESG_o_ (the environmental RH: 57.3~68.3%).

To investigate the mulching function of ESG_0_ at a rapeseed planting depth of 50 mm, the temperature and humidity changes in the potting soil were measured with a soil temperature and humidity sensor (ST-TR-WS 485, Shandong Measurement and Control Company, Zibo, China). The mulching results for ESG_0_ (0), ESG_0_ (200), and ESG_0_ (400) can be seen in Figure 10.

The following results can be obtained from Figure 9. The soil temperature and humidity of the mulched soil were all higher than that of the non-mulched soil. The relationship is ESG_0_ (400) ≥ ESG_0_ (200) ≥ ESG_0_ (0). The average humidity difference between ESG_0_ (400) and ESG_0_ (0) was 1.3%, and the average temperature difference was 0.95 °C. This is attributed to the improvement of the sealing property of soil pores by the water absorption and expansion of ESG_0_.

### 3.7. Characterization of Pot Experiment

**Figure 11:**
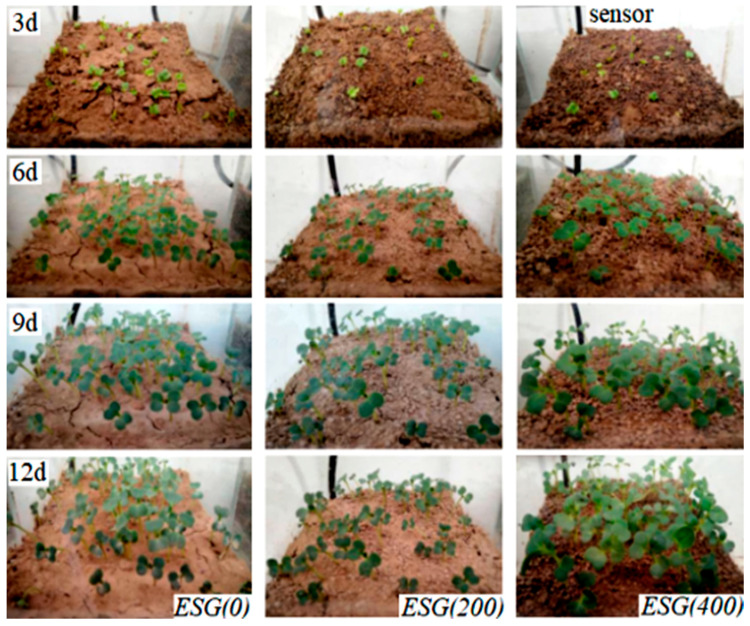
Growth of rapeseed under ESG mulching.

Figure 11 clearly shows the germination and growth of rapeseed after sowing. Almost all of the seeds had sprouted after growing for three days, but there were still distinct differences. The germination rates were ESG (0) > ESG (200) > ESG (400).

From the sixth to ninth days, all seedlings were able to grow well because there was an adequate supply of water in the soil. However, as the water evaporated, the growth difference became more pronounced, and the humidity difference increased (see Figure 10). In the pot covered with SEG (400), the soil maintained the necessary moisture to support the growth of vegetable seedlings. Since no water was added to the surface, the release of nutrients by SEG can be ignored. Potted plants covered with SEG(200) and SEG(0) showed that lack of water caused slow growth of vegetable seedlings. 

## 4. Conclusions

The soil in which plants grow is influenced by various factors, such as the surface structure of the soil itself, mulching status, soil moisture and temperature, air movement, and so on. Similar to other types of biomass mulching films, ESGs have the advantages of soil moisture conservation and environmental friendliness, despite their different morphology compared to previously studied biomass mulch films. However, encapsulated soil granules possess a unique mulching mechanism that allows them to roll freely into soil crevices and then absorb water and expand to cover the area. When used on rough soil surfaces, the encapsulated soil granules can be easily applied and a smaller amount can be used. The experimental results showed that optimal encapsulated soil granules can be produced by combining soil with a 10% PVA/PC-60 solution, and the mulching effect of the optimized encapsulated soil granules can be improved by adding an appropriate amount of PVA/PC-60. Using a small amount of PVA/PC-60 to create the wall material and using rural soil as the core of the encapsulated soil granules has significant practical significance for reducing costs and being environmentally friendly.

## Figures and Tables

**Table 1:** Chemical properties of the rural soil used in the study.

Element	C	O	Na	Mg	Al	Si	K	Ca	Ti	Fe
Wt/%	7.62	51.83	1.68	1.25	6.60	25.39	2.68	0.77	0.44	1.76

**Table 2:** Composition and synthesis of PVA/PC.

Film Name	Component Based on Effective Weight		
	PC Content (%)	PVA Content (%)	Urea Content (%)
PVA/PC-80	80	15	5
PVA/PC-60	60	35	5
PVA/PC-40	40	55	5
PVA/PC-20	20	75	5

**Table 3:** Water absorption rate of ESGs after immersion.

Time/h	2. Water Absorption (%)/Expansion Volume Rate (%)			
	3. ESG-20	4. ESG-15	5. ESG-10	6. ESG-5
7. 0	8. 0	9. 0	10. 0	11. 0
12. 3	13. 6.3/9.3	14. 55.9/84.5	15. 73.3/123.8	16. 78.2/133.7
17. 6	18. 12.2/15.8	19. 71.8/109.6	20. 90.1/156.1	21. 102.3/172.4
22. 9	23. 19.7/29.8	24. 98.6/165.8	25. 122.1//216.4	26. -
27. 12	28. 24.2/39.3	29. 103.5/196.1	30. 131.5/268.9	31. -

## Data Availability

The data in this paper are from the author’s experimental results. The data presented in this study are available on request from the corresponding author.
